# A closer look at neuron interaction with track-etched microporous membranes

**DOI:** 10.1038/s41598-018-33710-6

**Published:** 2018-10-19

**Authors:** Julian H. George, David Nagel, Sharlayne Waller, Eric Hill, H. Rhein Parri, Michael D. Coleman, Zhanfeng Cui, Hua Ye

**Affiliations:** 1 Institute of Biomedical Engineering, Department of Engineering Science, University of Oxford, Oxford, OX3 7DQ UK; 2 Aston Research Centre for Healthy Ageing, Life and Health Sciences, Aston University, Birmingham, B4 7ET UK

## Abstract

Microporous membranes support the growth of neurites into and through micro-channels, providing a different type of neural growth platform to conventional dish cultures. Microporous membranes are used to support various types of culture, however, the role of pore diameter in relation to neurite growth through the membrane has not been well characterised. In this study, the human cell line (SH-SY5Y) was differentiated into neuron-like cells and cultured on track-etched microporous membranes with pore and channel diameters selected to accommodate neurite width (0.8 µm to 5 µm). Whilst neurites extended through all pore diameters, the extent of neurite coverage on the non-seeded side of the membranes after 5 days in culture was found to be directly proportional to channel diameter. Neurite growth through membrane pores reduced significantly when neural cultures were non-confluent. Scanning electron microscopy revealed that neurites bridged pores and circumnavigated pore edges – such that the overall likelihood of a neurite entering a pore channel was decreased. These findings highlight the role of pore diameter, cell sheet confluence and contact guidance in directing neurite growth through pores and may be useful in applications that seek to use physical substrates to maintain separate neural populations whilst permitting neurite contact between cultures.

## Introduction

Microporous membranes are commonly used in many different types of cell culture application including the study of cell migration and the culture of cell sheets^1,2^. Differing from conventional dish cultures, membrane pores permit nutrient diffusion to the underside of the cell sheet and act as a reservoir for extracellular matrix and expressed factors. When cells are cultured as sheets, this can result in the polarisation of the cell soma^3^. Where cells are cultured on both sides of the membrane, pores allow for restricted connectivity between the cultures, for example between the astrocytes and endothelial cells in systems which model the blood brain barrier^4^. Porous membranes also play a key role in many of the emerging ‘lab-on-a-chip’ type culture systems, including lung^5^, stem cell differentiation^6^, blood-brain-barrier^7^, skin^8^, neural differentiation^9^, gut^10^, and liver^11^ applications.

The culture of neural networks directly on microporous membranes extends the study of neural cultures in flat bottomed dishes, enabling both neurons and neural processes to grow into and through membrane channels. In this way, the porous membrane acts as both a permissive substrate that supports neuron attachment and growth, and a restrictive substrate that limits the amount of connectivity between the culture surfaces on either side of the membrane. This arrangement also provides a two-layered culture environment, with both sides of the membrane accessible to conventional analysis using planar microscopy and dish-based assays.

Whilst porous membranes have long been used to support the viability of *ex-vivo* neural tissue slices^12^ allowing diffusion of nutrients and oxygen into the slice, the use of porous membranes as a culture substrate that facilitates the growth of neural networks is not well established. There is also a need to better understand how microporous substrates modulate network growth through investigation of the role played by pore shape, spacing, diameter, and channel length and structure. During neural network formation, the neurite growth cone is sensitive to surface features, such that nanostructures^13–15^ and microstructures^16–21^ encountered on the surface direct and modulate the growth of neural processes. Of the types of commercially available porous membranes, track-etched polymer membranes have flat culture surfaces and highly uniform diameter pores and channels^22^, making them especially appealing for the study of neural migration and neurite growth. Although neural cells have been found to migrate through track-etched micro-porous membranes with 8 µm diameter pores^23^, and smaller pore diameters (1 µm to 3 µm) have been found to restrict whole cell migration in the study of neurite outgrowth^24–26^, a systematic study of neuron and neurite response to track-etched membrane pore and channel diameter has not been reported.

In this report, fluorescence microscopy was used to investigate neuron and neurite growth through track-etched membranes with pore diameters ranging from 0.8 µm to 5 µm. Electron microscopy was used to gain a more detailed perspective of the interaction of neurites with pore edges. Based on the findings presented, an optimised pore diameter of 1.2 µm was used to demonstrate neurite growth through membrane channels between two separated neuronal populations.

## Results

**Figure 1:**
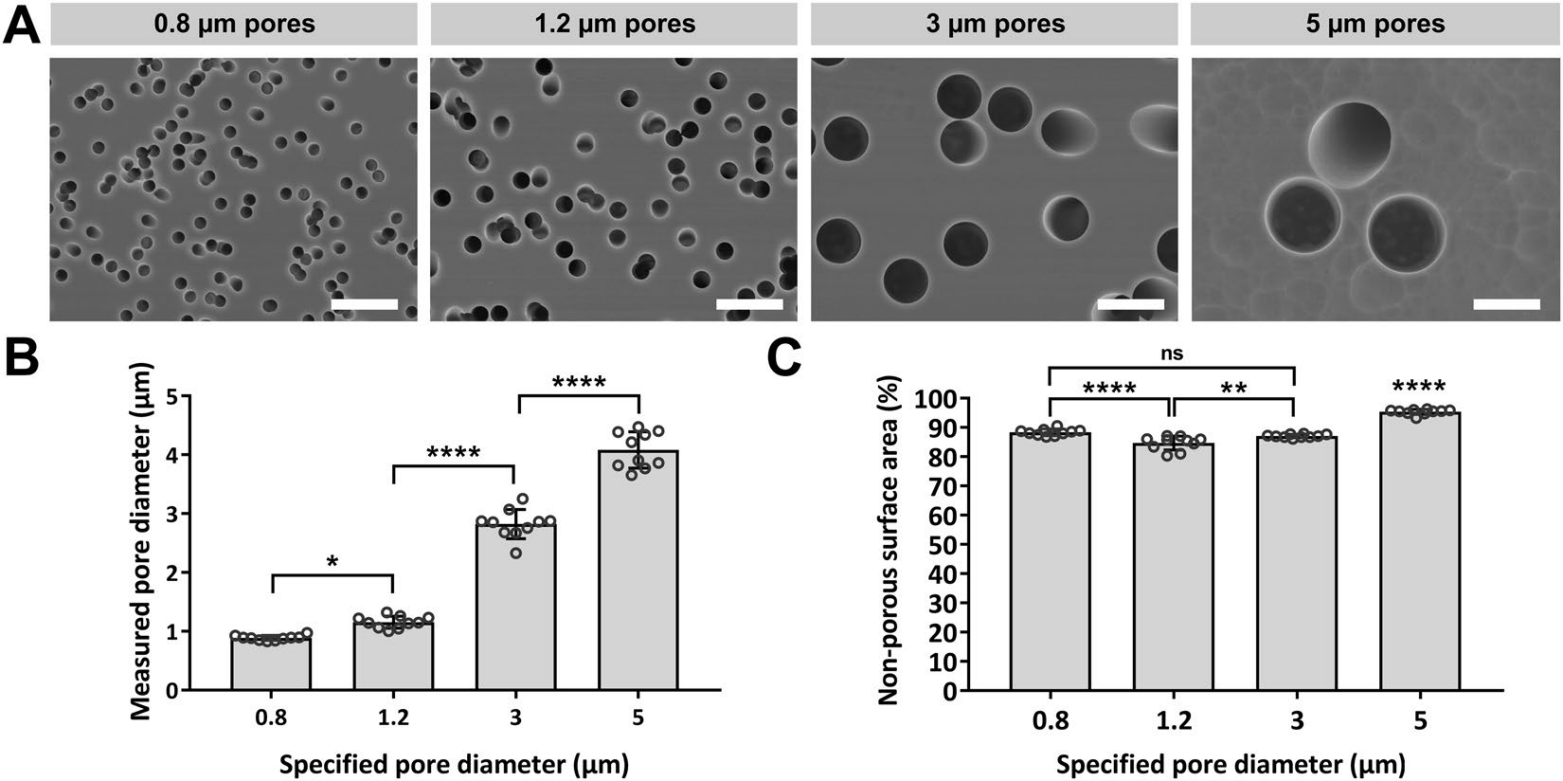
Scanning electron micrograph showing pore size, distribution and the uniformity of pore diameter for track etched microporous membranes. Plots show variance in pore diameter and non-porous surface area. Results are expressed as mean ± SD (n = 10) with dots representing single fields of view. p > 0.05 (*), p > 0.01 (**), p > 0.0001 (****), “not significant” (ns). *Scale bars (5 µm)*.

Track etched membranes have uniform pore diameters and straight channels that pass directly through the membranes. Analysis of scanning electron microscope images was used to confirm that pore distribution across the membrane surface, as well as porosity and pore channel diameters fell within manufacturer specified tolerances (Fig. 1). Channels originating at the surface were seen to take straight and direct paths through the 20–25 µm thick membranes and all membranes were found to have a large non-porous surface area available for cell culture, with 86 ± 3% non-porous surface area on the 0.8 µm, 1.2 µm and 3 µm pore diameter membranes, and 95 ± 1% non-porous surface area on the 5 µm pore diameter membranes.

**Figure 2:**
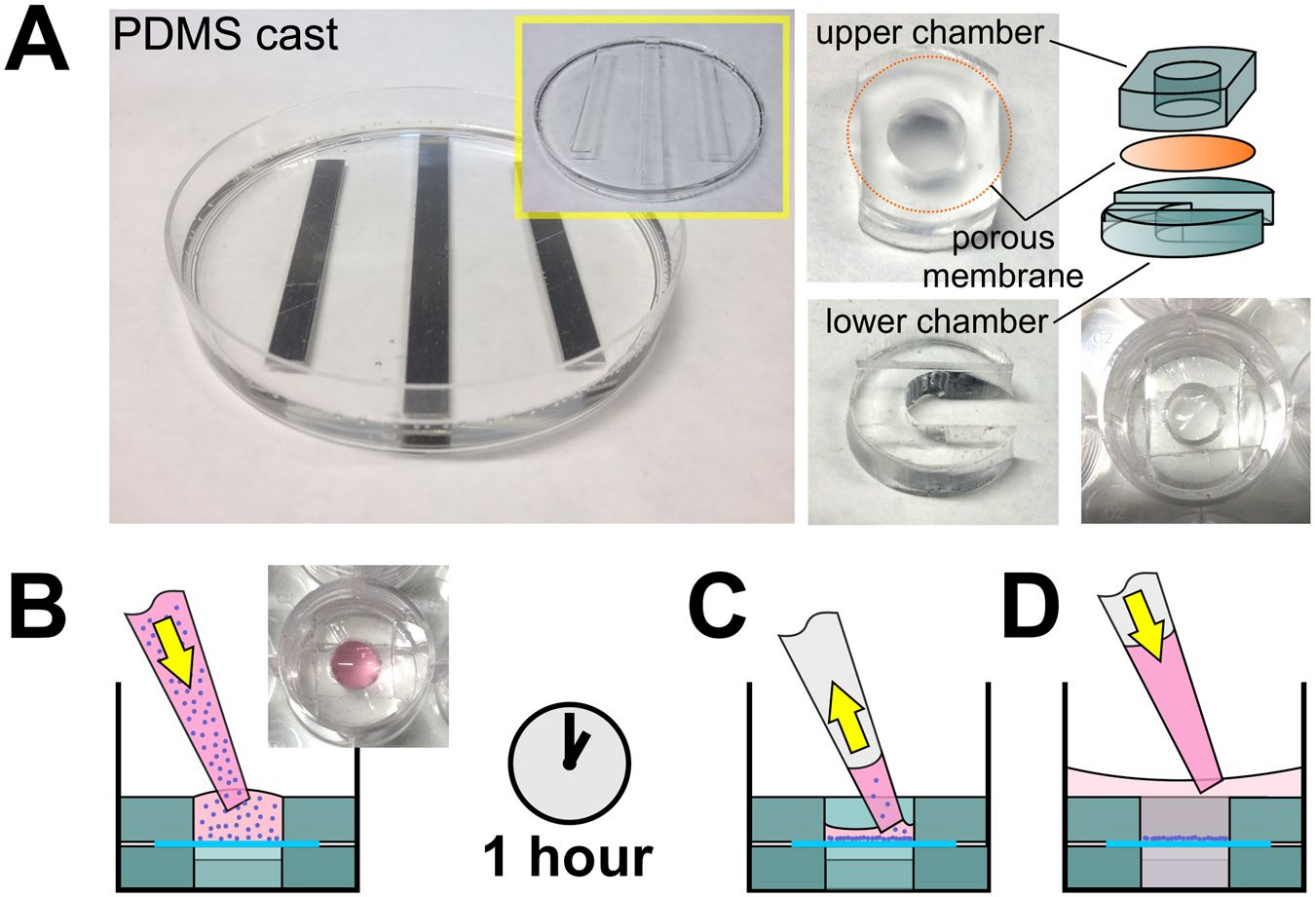
Schematic of microwell fabrication. (**A**) Fabrication of well-inserts showing the upper and lower chamber rings punched from PDMS cast in a 90 mm petri-dish. The porous membrane is held securely between the upper and lower chamber, inside a well of a 24-well plate during seeding and culture. (**B**) A cell suspension pipetted into the upper chamber is incubated on the membrane for 1 hour at 37 °C to allow cells to attach. (**C**) After this, non-attached cells are removed, followed by a PBS rinse to ensure only attached cells remain. At this point the membrane can be inverted and the underside seeded for two-layer culture. (**D**) Fresh culture medium covers both the bottom and top chambers during culture.

To investigate the constraint of neuronal cell migration through 0.8 µm, 1.2 µm, 3 µm, and 5 µm diameter micropores, 13 mm diameter track-etched membranes were held in place in wells of a 24 well plate using rings punched from a cast PDMS sheet (Fig. 2). In separate experiments, interaction of two different neuronal cell lines with the porous membranes was investigated. To ensure correct seeding density, neurons were seeded as a droplet into the central well above the membranes at high density (30 × 10^4^ cells/cm^2^) and incubated for one hour to allow for attachment before adding extra medium to the well (Fig. 2).

**Figure 3:**
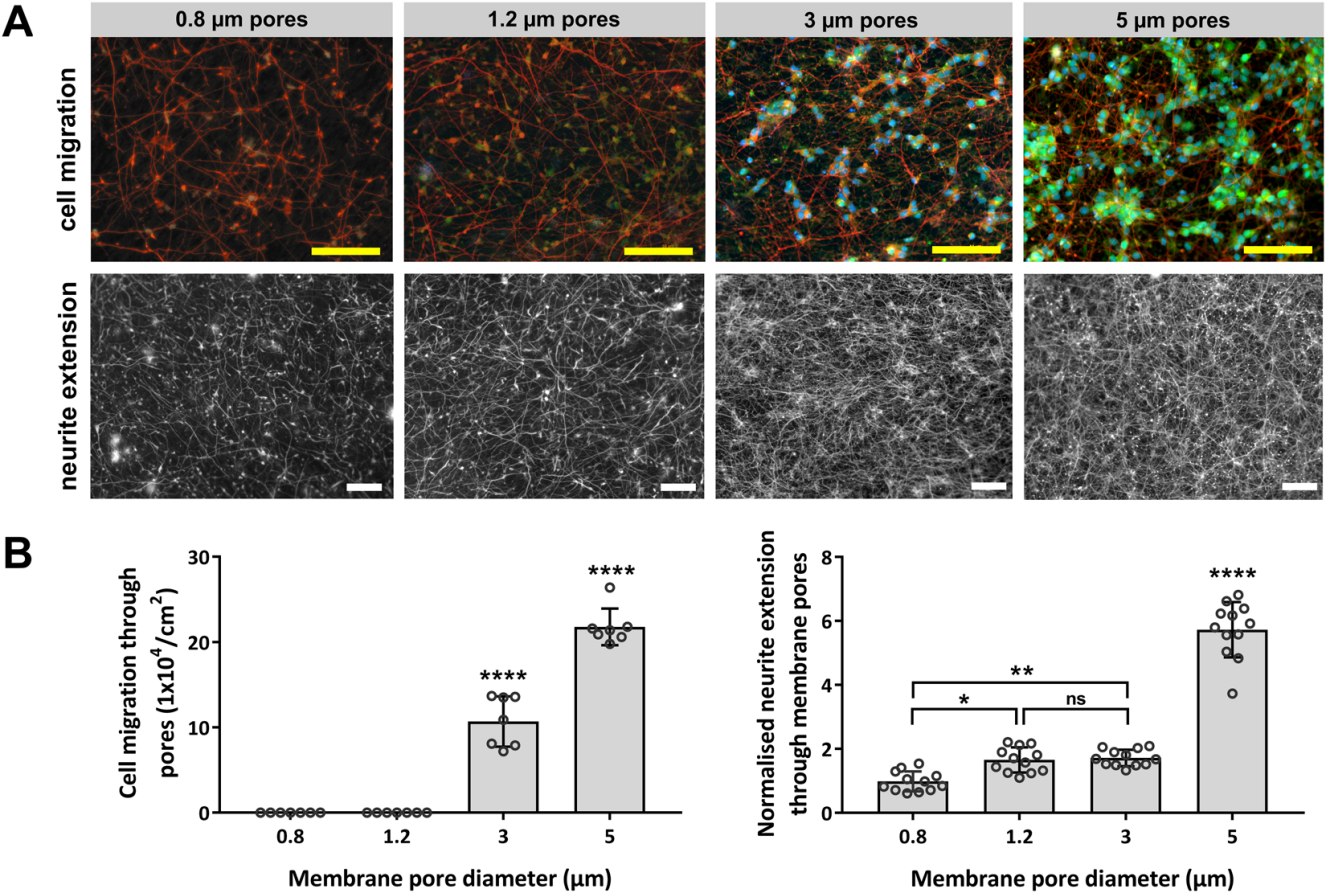
The effect of pore size on SH-SY5Y migration and neurite extension through the membrane. (**A**) Images show the lower non-seeded side of the microporous membranes after 5 days in culture, following removal of cells and neurites from the upper side of the membranes. (**B**) Cell migration plot shows counts of in-focus DAPI stained cell nuclei on the lower side of the membrane. Neurite plot shows the mean neurites on the lower side of the membrane in comparison to the 0.8 µm pore diameter membranes and after normalisation against differences in membrane porosity. Plots show the means ± SD with dots representing fields of view (cell migration: n = 7, neurites: n = 12). p > 0.05 (*), p > 0.0001 (****), “not significant” (ns). *Scale bar(50 µm)*.

After 5 days in culture, no cells had migrated through the 0.8 µm and 1.2 µm pore diameter membranes, whereas an average of 72 ± 20 cells/mm^2^ migrated through the 3 µm pore diameter membranes, and an average of 146 ± 15 cells/mm^2^ migrated through the 5 µm pore diameter membranes (Fig. 3), demonstrating that a pore diameter of less than 3 µm was required to adequately constrain neuron migration. Image analysis of βIII-tubulin immunostained neurons and neural processes was used to quantitate neurite extension through membrane pores and channels on the lower (non-seeded) side of the membranes. Neurite extension occurred through and onto on the lower side of all membranes for all pore diameters tested, with neurite density increasing in-line with pore diameter (Fig. 3). Neurite coverage on the 3 µm and 5 µm pore diameter membranes was in part attributed to neurons that migrated through the membrane.

**Figure 4:**
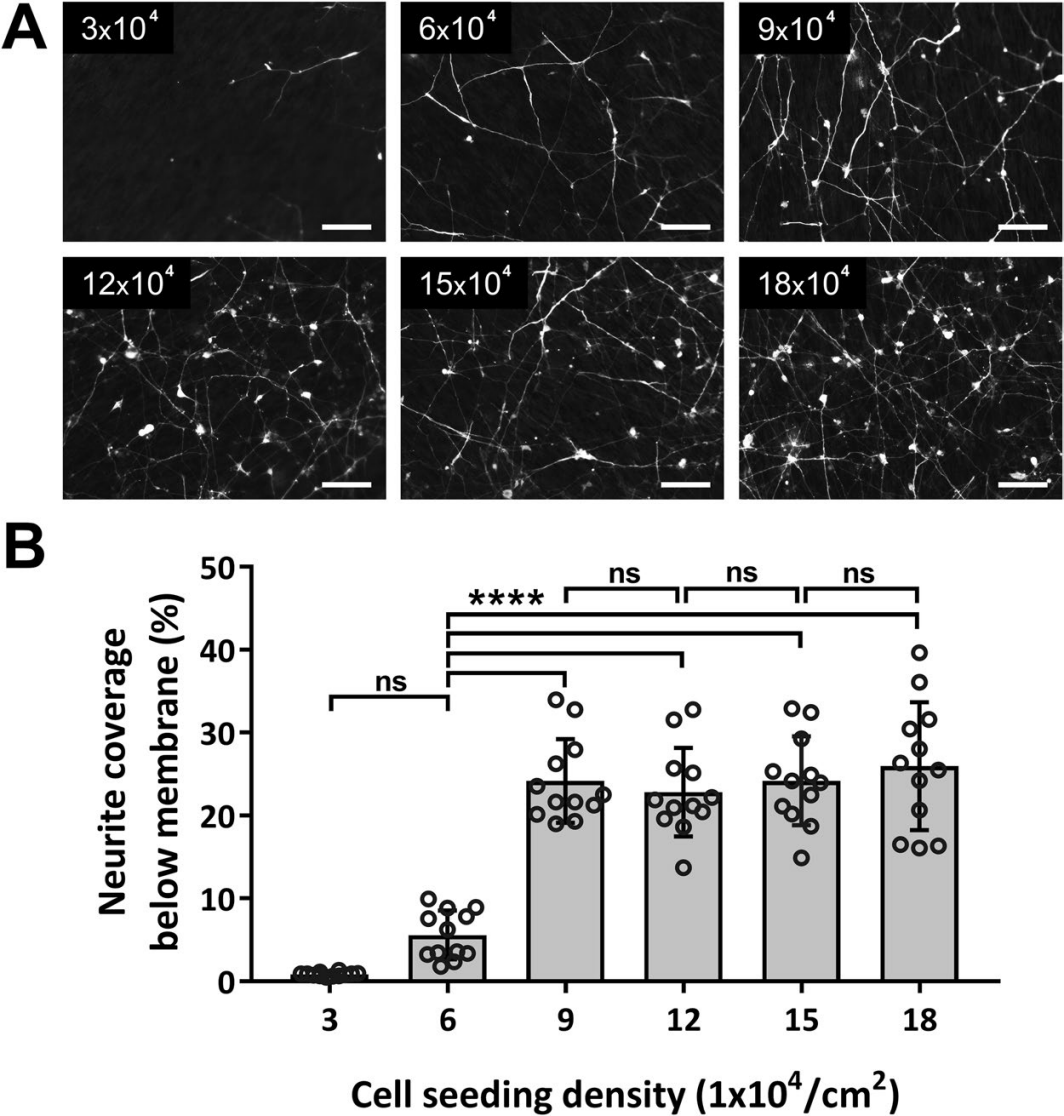
The effect of SH-SY5Y seeding density on neurite extension through 1.2 µm pore diameter membranes. (**A**) Images show microporous membranes after 5 days in culture with cells seeded at the marked densities on the upper sides of the membranes. The lower (non-seeded) side is shown, following removal of cells and neurites from the upper side (upper side not shown). (**B**) Image analysis was used to quantify the degree of neurite extension below the membranes in relation to seeding density. Plot shows mean neurite surface coverage ± SD with dots representing fields of view (n = 12). p > 0.001 (***), p > 0.0001 (****) and “not significant” (ns). *Scale bars (50 µm)*.

The SH-SY5Y neuronal cell model was chosen to study the effect of seeding density on neurite growth through pores, as SH-SY5Y neurons remain evenly distributed across the membrane over the 5 day culture period, facilitating image analysis, whereas we have found that other human neuronal cell types such as NT2/D1 (Supplementary Fig. S1) tend to form clusters on the membrane surface. The 1.2 µm pore diameter membrane was chosen for the seeding density study, having pore diameters that permitted the highest level of neurite outgrowth whilst preventing whole cell migration. Seeding density modulated the confluence of neuron cultures on the upper seeded side of the 1.2 µm membranes, which in turn modulated the amount of neurite growth observed on the lower side of the membrane, however, this relationship was non-linear (Fig. 4). At seeding densities greater than 9 × 10^4^ cells/cm^2^, a confluent layer of SH-SY5Y neurons formed above the membrane, whereas at lower seeding densities, the neural growth across the membrane was sparse (upper side not shown). Image analysis revealed that the amount of neurite coverage below the membrane was similar for all seeding densities above 9 × 10^4^ cells/cm^2^, and significantly reduced for lower seeding densities, which resulted in non-confluent cultures over the 5-day culture period. Very few neurites extended through membrane pores for seeding densities of 3 × 10^4^ cells/cm^2^.

**Figure 5:**
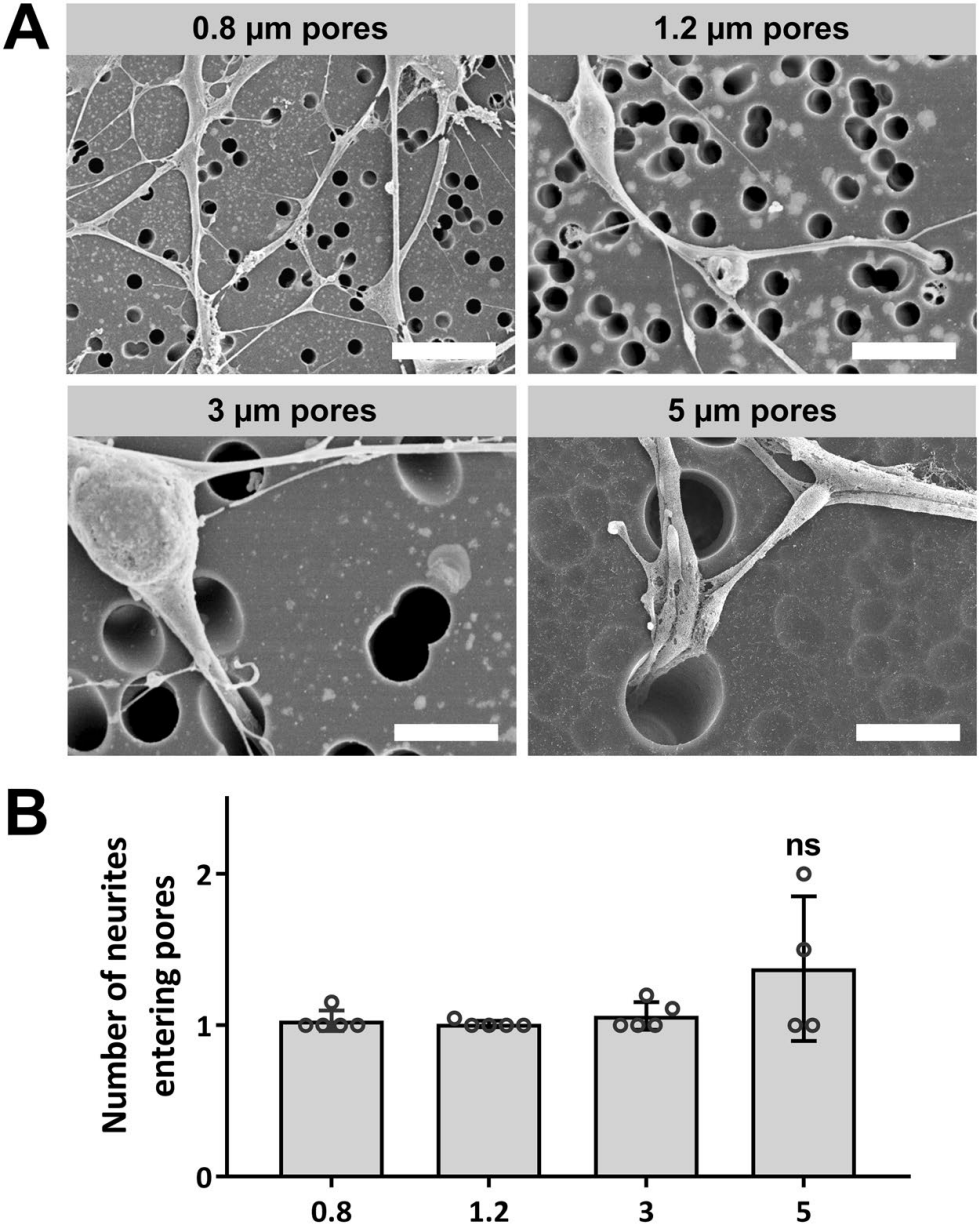
Representative SEM images of SH-SY5Y cultures on porous membranes. (**A**) Low seeding density cultures (1 × 10^4^ cells/cm^2^) show neurite interaction with 0.8 µm, 1.2 µm, 3 µm and 5 µm diameters pores. (**B**) Image analysis was used to quantify the number of neurites entering each pore, counted for one or more neurites. Plot shows means ± SD with dots representing fields of view (n = 5). “not significant” (ns). *Scale bars (5 µm)*.

**Figure 6:**
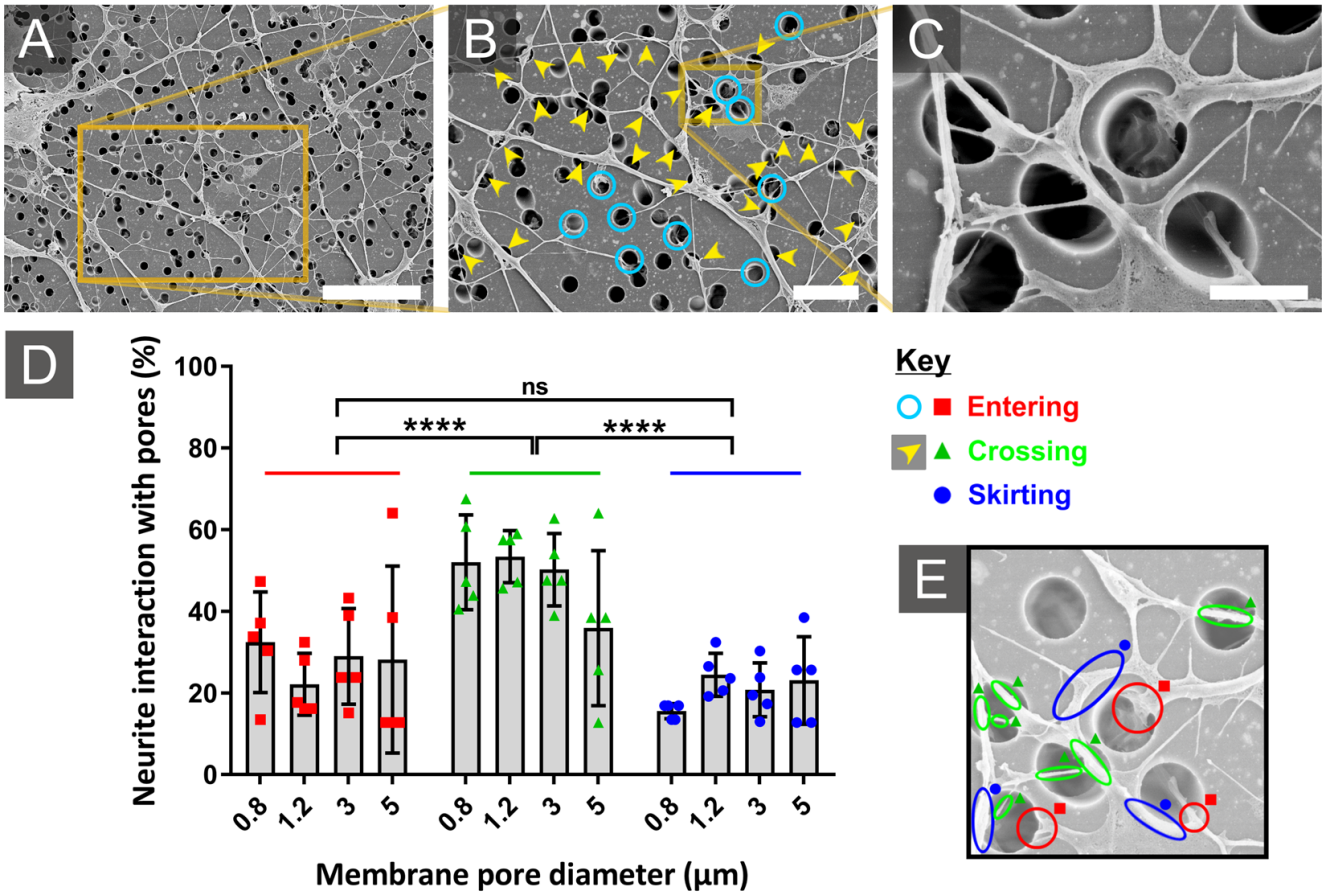
Representative SEM images of SH-SY5Y neuron interaction with porous membranes. (**A**) Overview of neurite interaction with 3 µm diameter pores. (**B**) Enlarged section revealing neurites crossing over pores (yellow arrows) and descending into pores (blue circles). (**C**) A further enlargement shows a neurite tracking around a pore edge, whilst another neurite climbs over the first to descend into the same pore. (**D**) Plot shows proportional neurite interaction with pores, counting neurites entering pores (red square marker), crossing pores (green triangular marker) and skirting the pore edge (blue circular marker). (E) Representative image with examples of interaction type highlighted. Plot shows means ± SD with dots representing fields of view (n > 4). p > 0.0001 (****) and not significant (ns). *Scale bars (A: 30 µm), (B: 10 µm), (C:3 µm)*.

Scanning electron microscopy was used to visualise neurite morphology and interaction with micropores. The width of the 0.8 µm and 1.2 µm diameter pores was found to be similar to the width of the more mature neurites that extended through pores, and mean growth through the membrane was found to be restricted to one neurite per pore/channel (Fig. 5). The 3 µm and 5 µm diameter pores were large enough to permit multiple neurites to pass through each pore/channel. Examination of low density neural cultures on 3 µm pore diameter membranes revealed that, despite the presence of neurites directly encountering pores, neurites more often extended directly over a pore than enter into the underlying channel (Fig. 6). Upon encountering the edge of a pore, neurites and migrating cells were also found to change direction, suggesting that the growing neurite was sensitive to the pore edge boundary (Supplementary Figs S2–S6).

**Figure 7:**
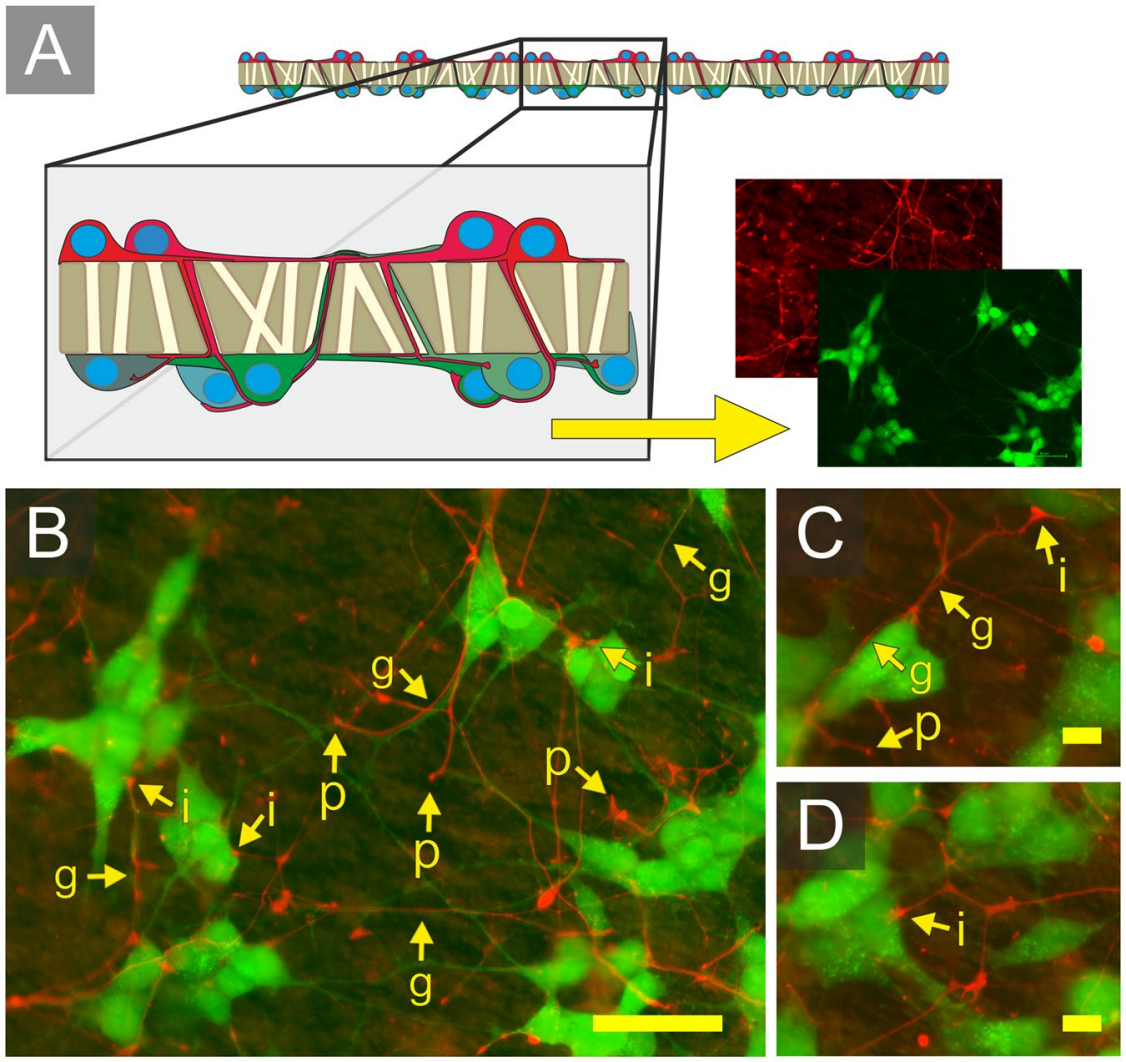
Neurite growth of spatially separated SH-SY5Y neural populations across a 1.2 µM membrane. (**A**) Schematic demonstrating the membrane separating EGFP expressing (lower layer) and mCherry expressing (upper layer) neurons. (**B**–**D**) Images of the lower membrane. Arrows highlight: (p) the upper layer neurites emerging through pores, (g) upper layer neurites co-localised with lower layer neurites, and (i) upper layer neurites co-localised on lower layer neural soma. *Scale bars (B: 50 µm) and (C, D: 10 µm)*.

To demonstrate separation of two distinct neural networks across a porous membrane, mCherry expressing red neurons were seeded at high density to form a confluent cell layer on a membrane with the previously determined optimal pore diameter of 1.2 µm (Fig. 7). The example culture demonstrates an application that combines the use of membrane pore size (Fig. 3) and seeding density (Fig. 4) to facilitate neuron compartmentalisation. To enable clear visualisation of projecting mCherry expressing neurites, EGFP expressing green neurons were seeded at lower density on the opposite face of the membrane to form a lower density network. After 5 days in culture it was possible to visualise neural processes from the mCherry expressing neurons extending through pores to the opposite face of the membrane containing EGFP expressing neurons.

## Discussion

Culture substrates that contain micro-channels can be used to isolate groups of neurons within a cultured neural network whilst allowing connections to form between these groups via neurite extension through channels. Using this approach, it is possible to generate compartmentalised neural culture models that support stable, long term growth of engineered network architectures. The ability to physically constrain neural processes inside channels is increasingly being used in culture systems to guide and direct neurite growth, commonly through the use of microstructured constructs placed onto culture surfaces to create an array of tunnels that are horizontally in line with cell growth and neurite extension^27–30^. Alternatively, culturing neurons on the surface of a porous membrane enables neural interaction with a dense array of channels that descend into the culture surface, such that a neurite growing across the surface would need to change direction to extend down into the vertical channel. This second approach is relevant to applications where two culture layers are connected via microchannels that extend from the underside of the culture. The results described are relevant to this arrangement and demonstrate that defined pore sizes can be used in culture to restrict neuron migration whilst permitting neurite extension into membrane channels extending down from the culture surface. Cell migration through pores is known to be restricted by cell size, and more specifically by the size, shape and stiffness of the cell nuclei^31,32^. In this study, cells were able to migrate through 3 µm diameter channels, but unable to migrate through the smaller 1.2 µm diameter channels (Fig. 3). However, the nuclei diameter for the SH-SY5Y neurons in this study was measured as 7.3 ± 1 µm (n = 40), demonstrating that cells were able to undergo a large degree of conformational change during microchannel migration. We have found similar results when culturing the neuronal NT2/D1 human cell line on laminin coated porous membranes, with no neural migration through pores smaller than 1.2 µm in diameter, and variable cell migration for 3 µm and 5 µm diameter pores (Supplementary Fig. S1), however, quantification of cell migration and neurite growth of NT2/D1 neurons proved challenging due to the growth characteristics of the NT2/D1 line. Whilst the differentiated NT2/D1 morphology resembled NT2/D1 growth on other tissue culture substrates and allowed visualisation of projecting neurites, NT2/D1 neurons clustered into large groups over the 5 day culture period, which resulted in variable levels of cell density across the membrane. In contrast, SH-SY5Y neurons did not form clusters. Migration of other human cell types through micropores is also likely to be constrained by similar channel diameters. For example, human epithelial cells have been found to have a similar migration response, where nuclei conformational change supported migration through 3 µm diameter pores, but not though smaller pores^33^.

Pore diameter was also shown to modify the degree of neurite extension through channels, with neurite coverage under the membrane significantly reduced for 0.8 µm diameter pores in comparison to 1.2 µm diameter pores (Fig. 3B). Analysis of SEM images demonstrated that predominantly single neurites entered pores from the SH-SY5Y neurons, even for larger 3 µm and 5 µm diameter pores that could accommodate multiple neurites (Fig. 5). The SEM images analysed were from low density neural networks and it may be that the occurrence of multiple neurites entering a single pore will increase for higher density neural networks. Neurite extension of rat adrenal pheochromocytoma (PC12) cells through pore diameters as small as 0.4 µm has previously been reported^34^, however, in this study it was found that the 0.8 µm channel diameters constrained the overall coverage of neurites, and by association the length of neurites emerging below the membrane, in comparison to neurites emerging through the 1.2 µm pore diameter membranes (Fig. 3). This was despite the 0.8 µm pore diameter membrane having a greater number of pores/cm^2^ than the 1.2 µm pore diameter membrane (Fig. 1). It was thought that the smaller 0.8 µm channel diameter may have modulated gross transport along the neurite, which would in-turn reduce the rate of neurite growth downstream of the channel exit, frustrating the formation of the neurite cytoskeleton.

Seeding density above the membrane was found to have a significant impact on the degree of neurite extension through the selected 1.2 µm diameter pores and the extent of neurite coverage below the membrane (Fig. 4). The seeding of a greater number of cells naturally increases the probability of cell-pore interactions, however, the relationship between seeding density above the membrane and neurite growth below the membrane was non-linear. For confluent cultures, neurons will be positioned directly above pores. For non-confluent cultures, neurons must extend neurites up to and over the edge of a pore to descend into the pore channel. In these cultures, neurites were seen to typically extend further than 100 µm from neural soma in a pattern that was highly exploratory. Despite this, pore uptake from non-confluent cultures was significantly lower than expected.

High magnification SEM images of neurite interaction with pores revealed that neurites appeared to be sensitive to pore edges (Fig. 6). For a neurite to grow through a pore channel, the growth cone at the tip of the neurite must contact and adhere to the inside wall of the channel and extend through the membrane before extending from the channel and onto the lower face of the membrane. SEM imaging revealed many cases where neurites diverted away from pore edges and spanned across pore channels (Fig. 6B), and cells were observed approaching pores and then retreating (Supplementary Figs S2–S6). Image analysis revealed that whilst the number of neurites entering pores and skirting pore edges was similar for all pore diameter membranes, significantly more neurites were observed to cross directly over the top of the micropores, spanning the pore mouth (Fig. 6D). It has previously been shown that neurites will span large gaps, and a process has been described by which neurites can bridge 60 µm wide grooves by simultaneously extending along opposing walls of the groove^35^. In the case of spanning micropores, it is more likely that a simpler process is taking place. When navigating a surface, neurites will form sporadic attachment points on the surface. When the growth cone of the neurite extends to explore the culture surface beyond a pore, the trailing neurite will be pulled taught across the pore. Diversion away from a pore may occur when the growth cone, which explores the surface via lamellipodia and filopodia, extends preferentially within the same plane rather than bending over an edge to extend into a different plane. This type of behaviour follows from the tensegrity model of the cytoskeleton^36^, where the components that make up the neurite cytoskeleton may resist sharp changes in culture substrate direction^37,38^. Contact guidance, caused by forces imposed on filopodia by the underlying surface topography, is also known to reshape cytoskeleton actin filaments, matching the cytoskeleton extension to the surface topography^39^. This could cause neural processes to extend along or withdraw from pore edges, rather than cross over the angular surface into the channel below.

Based on these results, an optimised pore diameter of 1.2 µm was selected to demonstrate the formation of neurite connections between two neural networks seeded with differentially labelled neurons at different densities on either side of a porous membrane. Both networks were visible, accessible, and compartmentalised by the membrane, showing a preference for directional neurite extension (from high to low seeding densities) and no cell migration occurred between the two networks (Fig. 7). It is expected that this approach could be used to enable highly connected networks to form between two cultures that need to be accessed and investigated separately. Future experiments will investigate the synaptic connectivity and electrophysiological activity between separated neuronal cultures.

## Conclusions

The architecture of cultured neural networks can be constrained by engineering microscale channels into culture substrates. The growth of stable neurite connection pathways inside these channels during long term cultures is a viable approach for producing complex network structures. However, greater understanding is needed as to how neurite uptake and subsequent neurite extension is modulated by channel diameter, length and the shape of the surface pores at the entrance and exit to channels in culture substrates. In this report it was demonstrated that channel diameters can be used to constrain cell migration whilst facilitating neurite connections between neurons in culture. Specifically, a 1.2 µm channel diameter was found to be ideal for restricting the migration of neural soma whilst permitting extensive neurite growth and interaction between two distinct neural networks. A contact guidance effect on the membrane surface may have reduced the likelihood that cellular processes entered pore channels, such that a neurite growing from a neuron in a non-confluent culture would be less likely to extend into the channels that it encountered when exploring the surface of the culture substrate. For non-confluent cultures, tailoring the shape of pore entry geometry may provide a way to use contact-guidance to control the direction of neurite growth into membrane channels.

## Methods

### Chamber manufacture and membrane preparation

Elastomer rings were punched from cast elastomer sheet to securely hold the porous membrane in wells of a 24-well plate (Fig. 2). Poly(dimethylsiloxane) (PDMS) (Dow Corning Sylgard 184, Farnell, UK) consisting of an elastomer base and curing agent mixed at a ratio of 10:1 (wt/wt) was poured into 90 mm petri-dishes containing 1 mm thick aluminium strips to generate channels. After curing at 90 °C for 4 hours, rings with an inner diameter of 9 mm and an outer diameter of 15.5 mm were cut from the cast sheets using a hole punch and autoclaved at 121 °C before use. Overlapping notches (3 mm diameter) were punched out of the outer surface of the upper rings and inner surface of the lower rings to allow for mixing of medium between the upper and lower chambers. To prepare the membranes for culture, Isopore® track-etched polycarbonate membranes (13 mm diameter; 20–25 µm thick; 0.8 µm, 1.2 µm, 3 µm, 5 µm, 8 µm pore size; Millipore, UK) were sterilised by immersion in a mix of 70% ethanol in sterile distilled water for 1 hr, air-dried and then exposed to oxygen plasma in a plasma cleaner (ATTO; Heiniker Plasma, UK) at 40 kHz with 120 W of power for 60 seconds to decrease surface hydrophobicity. Membranes were then incubated at 37 °C for 2hrs with 1 ml of 1% (w/v) poly-D-Lysine (Sigma-Aldrich, UK) in phosphate buffered saline (PBS; Life Technologies, UK), then rinsed in PBS and secured into the custom made PDMS chambers for seeding.

### SH-SY5Y neuron culture

The SH-SY5Y human neuroblastoma cell line was obtained from the European Collection of Cell Cultures (cat. no: 94030304) and routinely cultured in complete media composed of RPMI 1640 with stable glutamine (GE Healthcare, UK), supplemented with 0.1 mM nonessential amino acids (GE Healthcare, UK), 10% (v/v) foetal bovine serum (FBS; Gibco, UK) and 1% penicillin (100 U/mL) with streptomycin (100 mg/mL) (GE Healthcare, UK). Cells were incubated with 5% CO_2_ in a humidified atmosphere at 37 °C. Medium was exchanged every 2–3 days, and cultures were split when 80% confluent. Cells for monolayer culture were differentiated in T75 flasks (Corning, USA) for 7 days through exposure to 10 µM all-trans-retinoic acid (RA; Sigma-Aldrich, UK) and harvested using 1x Trypsin (0.5% w/v) with EDTA (0.02% w/v) (GE Healthcare, UK) for seeding onto membranes. To investigate the effect of pore diameter on cell migration and neurite growth through the membrane, cells were seeded at high density (30 × 10^4^ cells/cm^2^) to obtain confluent cell layers. To investigate the effect of seeding density on neurite growth through the membrane, cells were seeded at lower densities (20 × 10^4^, 15 × 10^4^, 12 × 10^4^, 9 × 10^4^, 6 × 10^4^, and 3 × 10^4^ cells/cm^2^) and at very low density (1 × 10^4^ cells/cm^2^) to reveal neurite interaction with pores. In all investigations, cells were cultured on membranes for 5 days. To stimulate neurite outgrowth whilst attached to membranes, cells previously differentiated in RA were cultured in Neuronal Base Medium P (GE Healthcare, UK) supplemented with 1% PS, 2% (v/v) Neuronal Stem Cell Supplement (GE Healthcare, UK), 10 ng/ml nerve growth factor (NGF; Sigma-Aldrich, UK) and 50 ng/ml brain-derived neurotrophic factor (BDNF; PeproTech, UK).

### Neuron transduction

To distinguish SH-SY5Y cells in two-layer culture, cells that were to be used in the lower layer were transduced with lentiviral constructs driving constitutive expression of enhanced green fluorescent protein (EGFP) under the control of the human elongation factor-1 alpha promoter (EF-1α). Cells to be used in the upper layer were transduced with lentiviral constructs encoding mCherry fluorescent protein. Briefly, the sequence encoding EGFP or mCherry was placed downstream of the EF-1α promoter in the lentiviral expression vector pLenti6.4/R4R2/V5-DEST (Life Technologies, UK). Viral particles were quantitated using the QuickTiter™ Lentivirus Titer Kit (Cell Biolabs, inc., San Diego) and used to transduce SH-SY5Y cells in 25 cm^2^ flasks at a multiplicity of infection (MOI) of one viral particle per cell. After transduction for 12 hours, flasks were washed three times with media and then allowed to recover for 6 hours. Subsequently cells were washed with PBS and detached from the flasks using 0.5% trypsin EDTA (PAA, UK). Cells were washed by centrifugation, and resuspended in media. Transduced cells were seeded into 6-well cell culture plates (Corning, USA) with an initial seeding density of (200 cells/cm^2^) in the first well, followed by a serial 1:2 dilution across the remaining wells. Cells were allowed to recover for 24 hours prior to selection by the addition of Blasticidin (Life Technologies, UK) to the medium at a final concentration of 3 µg/ml. After selection, Blasticidin resistant colonies were isolated by trypsinisation using glass cloning cylinders (Sigma-Aldrich, UK), expanded and subsequently maintained in SH-SY5Y media containing Blasticidin (3 µg/ml).

### Two-layer culture

EGFP expressing cells were seeded at lower densities of (2 × 10^4^ cells/cm^2^) on top of each membrane and incubated for 1 hr, after which time most cells had attached. Non-attached cells were removed by washing with fresh media, and to facilitate two layer cultures, the upper PDMS ring was removed, and the membrane inverted and replaced such that attached cells were positioned beneath the membrane. The top PDMS ring was then replaced and pushed securely into place to hold the membrane for the second seeding. Cells expressing mCherry protein were seeded on top of the membrane at a higher density of 20 × 10^4^ cells/cm^2^ and the dual layer culture was incubated for a further 5 days, changing media above the membrane every 2 days.

### Fixation, staining and preparation for imaging

Prior to fixation, membranes were removed from wells and gently washed in PBS. Cells were fixed by incubation with 4% paraformaldehyde (PFA; Sigma-Aldrich, UK) in PBS at 4 °C for 15 minutes. Fixed membranes were washed three times in PBS and stored for up to one week in PBS at 4 °C. For dual layer cultures, direct imaging of the expressed fluorescent protein was used to visualise cells and neurites. For all other cultures, neurites were imaged after immunostaining of βIII tubulin. Prior to staining, cells were blocked and permeabilised at room temperature for 1 hour in PBS containing 10% bovine serum albumin (BSA; Sigma-Aldrich, UK) and 1% Triton X-100 (Sigma-Aldrich, UK). For βIII tubulin staining, membranes were inverted and placed into wells of a 24-well plate, such that neurite outgrowth was exposed above the membrane. The membranes were incubated overnight at 4 °C with mouse monoclonal anti-βIII tubulin antibody (R&D, UK), diluted 1:1000 in staining buffer (1% BSA and 0.1% Triton X-100 in PBS). After incubation membranes were washed three times in staining buffer and incubated at room temperature for 2 hours with Alexa Fluor 568 goat anti-mouse IgG (Life Technologies, UK) diluted 1:500 in staining buffer.

Removal of cells from the upper seeded side of the membrane is a technique commonly used in migration assays that reduces background florescence signal and permits more rapid and accurate imaging of the lower non-seeded side of the membrane using widefield microscopy. Excess liquid was removed by blotting with filter paper, the membrane was held taught and the top side wiped thoroughly using a PBS soaked cotton swab to remove cell bodies and neurites from the seeded side of the membrane. Membranes were gently washed three times in PBS and mounted onto slides with mounting medium (Vectashield, Vector, UK) containing the blue fluorescent nuclear dye 4′,6-diamidino-2-phenylindole (DAPI).

### Image acquisition and analysis

SEM imaging of cells cultured on membranes was carried out after fixing cells in 4% PFA. Membranes were dehydrated in a graded series of ethanol concentrations in PBS (25%, 50%, 75%, 80%, 90%, 2 × 100%) for 5 minutes per rinse followed by chemical drying using two rinses in 1,1,1,3,3,3-hexamethyldisilazane (HMDS, Sigma-Aldrich, UK) for 5 minutes each, prior to air drying. Membranes imaged without attached cells did not require dehydration. All samples were sputter coated with platinum and imaged under vacuum using a field emission SEM (JSM-840F; JEOL, UK) operating at an accelerating voltage of 5 kV.

Fluorescently stained and fluorescent protein expressing cells were imaged using a cooled CCD camera (EXI blue; QImaging, UK) attached to an inverted microscope (Eclipse Ti, Nikon) with LED illumination (pE-2; CoolLED, UK). Manual counting of in-focus DAPI stained cell nuclei below the membrane was used to estimate cell migration. Estimates for the area of neurite coverage below the membrane were obtained from image analysis of grayscale fluorescence intensity images of βIII tubulin stained cultures. For each field of view, counting of pixels with a fluorescence signal above a threshold determined using a default thresholding process (ImageJ Software, National Institute of Health, USA) was used to obtain the approximate coverage of βIII tubulin comparison to the total field area. Studies were performed in triplicate, with 7 or more fields imaged per sample (10x lens, each field measured 0.6mm^2^). To ensure a fair comparison, neurite growth through each membrane was normalised by dividing the calculated neurite area per field against the calculated porous area per field and then adjusting results relative to the calculated neurite growth through 0.8 µm pores. Characterisation of membrane pore size and porosity was performed semi-automatically using routines written in ImageJ software. Briefly, to calculate porosity a binary threshold was applied to SEM images using the default ImageJ algorithm and the black pixel area in 10 fields (representing the pores) was divided against the total pixel area to calculate the porous and non-porous ratios. To determine pore size, lines were manually draw onto the SEM images and the pixel length of the lines was used to calculate the average pore diameter, calibrated against a given scale. The results averaged across fields a number of fields (n > 10) with at least 3 pores per field (n > 30). To determine neurite interaction with pores, counting occurrences was performed using the imageJ Cell Counter plugin across multiple fields (250 µm^2^; n > 4), with at least 7 interactions counted on average per field (n > 28).

### Statistical analysis

Statistical analyses were performed using Prism Version 7.05 (GraphPad, San Diego, CA). One-way ANOVA (Figs 1 and 3–5) and two-way ANOVA (Fig. 6) was used to compare significance across groups, with the Brown-Forsythe test used to ensure that variance was similar between the groups tested and post-hoc analysis performed using the Tukey method. Results are expressed as mean ± SD with *, **, *** and **** representing p < 0.05, p < 0.01, p < 0.001 and p < 0.0001 respectively. A p value < 0.05 was considered statistically significant.

## Electronic supplementary material

Supplementary Information

Figure S3

Figure S4

Figure S5

Figure S6

## Data Availability

Plasmids and protocols used in this work are available on request.
