# Supplementary material for: A closer look at neuron interaction with track-etched microporous membranes
Source: Sci Rep. 2018 Oct 19;8:15552. doi: 10.1038/s41598-018-33710-6 (PMC6195627; doi:10.1038/s41598-018-33710-6)
Supplement: Supplementary file 1 — Supplementary Information [file 41598_2018_33710_MOESM1_ESM.docx]

**A closer look at neuron interaction with track-etched microporous membranes

Julian H George^1,+^, David Nagel^2,+^, Sharlayne Waller^1^, Eric Hill^2^, H Rhein Parri^2^, Michael D Coleman^2^, Zhanfeng Cui^1^, Hua Ye^1,*^**

^1^Institute of Biomedical Engineering, Department of Engineering Science, University of Oxford, Oxford, OX3 7DQ, UK

^2^Aston Research Centre for Healthy Ageing, Life and Health Sciences, Aston University, Birmingham, B4 7ET, UK

**^+^**These authors contributed equally to this work

**^*^**Corresponding author: H.Y. ([hua.ye@eng.ox.ac.uk](mailto:hua.ye@eng.ox.ac.uk)) Tel: +44 (0)1865 617689, Fax: +44 (0)1865 617701

**Supplementary Information**

**Supplementary Figures**


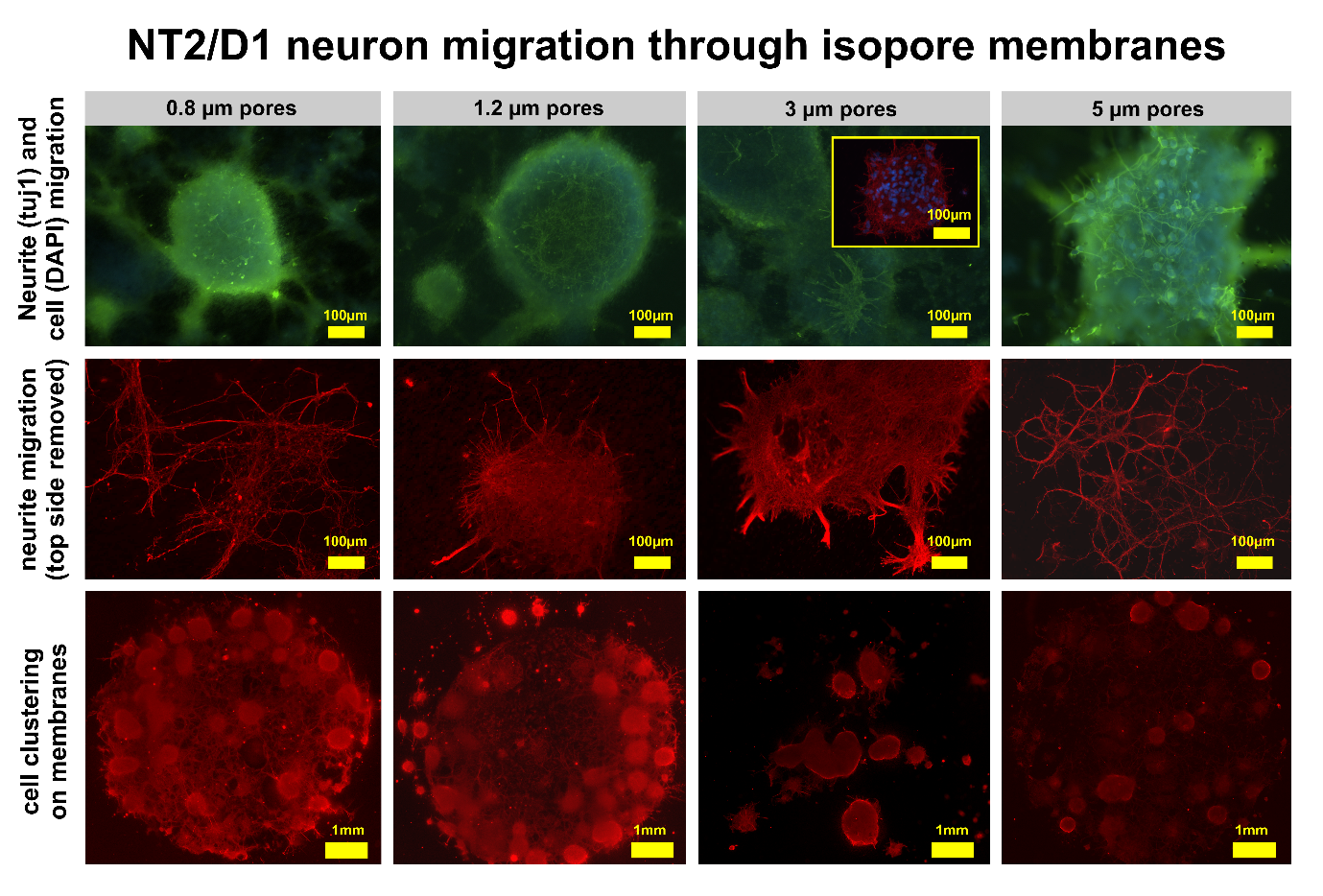


**Figure S1:** The growth of the NT2/D1 neuronal cell line through microporous membranes was also investigated. As neurons matured, it was found that they formed clusters on the Laminin coated membrane surface, with neurites penetrating through the membranes in the middle of the clusters. This significantly increased variability of neurite and cellular growth through the bulk of the membranes, and for this reason it was not possible to perform quantitative analysis on the data gathered. It was also found that no cells penetrated through the 0.8µm and 1. µum pore diameter membranes, whilst cells variably penetrated through the 3µm and 5µm pore diameter membranes. The top row shows neurons stained for Tuj1 (green) and DAPI (blue), focused on the lower side of the membrane with cellular clusters not removed from the upper side. For the 3µm image, whilst cells did not penetrate through in one study, they did in subsequent studies (inset with Tuj1 (red) and DAPI (blue)). The middle row depicts neurite growth under the membrane with the top layer removed. The lower row of images depicts an overview of the membranes showing typical neural clustering.  *Scale bars (20µm for the upper two rows; 1mm for the lower row)*.


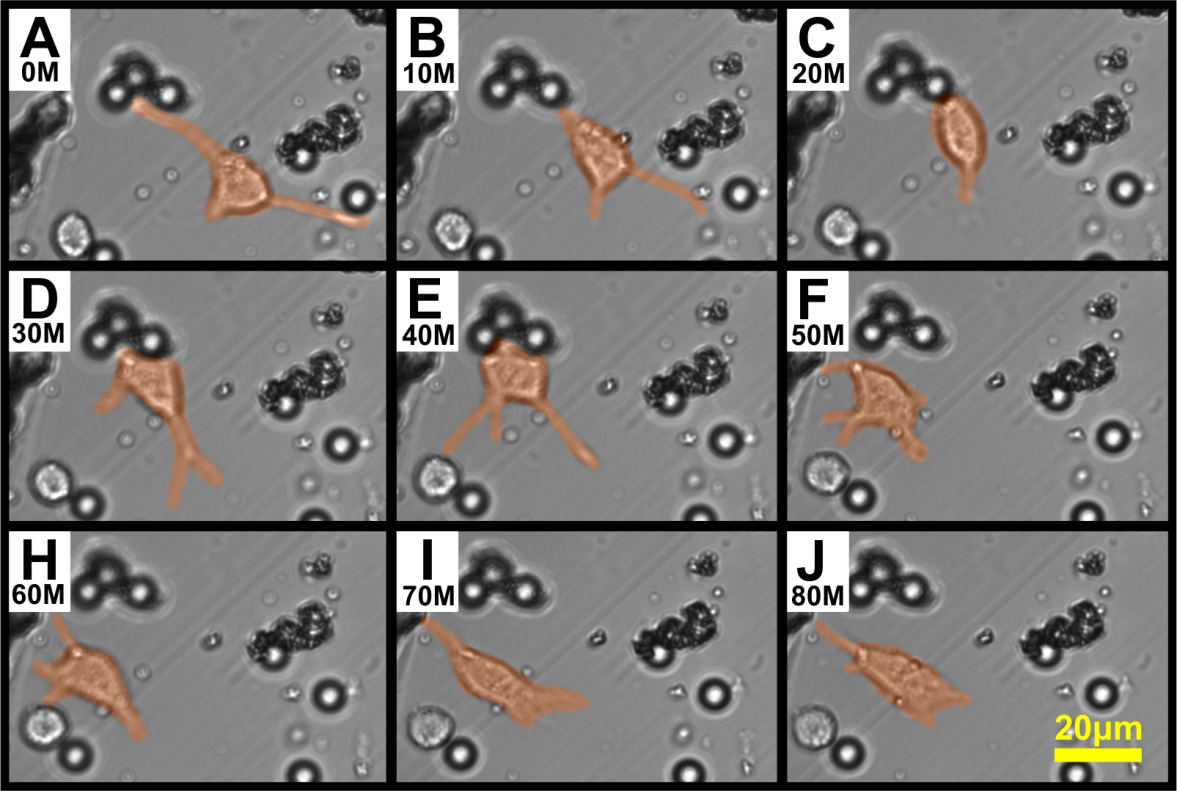


**Figure S2. Live imaging sequence of an SH-SY5Y cell on a microporous membrane.**  The image sequence (A-J) shows representative behaviour of an SH-SY5Y neuron approaching a cluster of 8µm diameter pores (top left), turning and then migrating away from the pores. Images were taken at 10 minutes intervals and are false coloured to highlight cell interaction. Full video shown in Fig S3. *Scale bar (20µm)*

**[Videos – please see supplemental video files]**

**Figure S3. [Figure-S3 (Ye).mp4] Video of the image sequence in Fig S2, depicting the live imaging of an SH-SY5Y cell on an 8µm pore diameter microporous membrane**. The SH-SY5Y cell approaches a cluster of micropores (top left of the frame) and after coming into contact with the pore edges, the cell changes its direction of movement, moving away from the micropore cluster. Approximate playback speed of 300x, with original video recorded over a 4-hour period. *Scale bar (10µm).*

**Figure S4. [Figure-S4 (Ye).mp4] Video shows live imaging of an SH-SY5Y cell on an 8µm pore diameter microporous membrane**. The SH-SY5Y cell is surrounded by a number of micropores and after coming into contact with each pore, the cell changes its direction of movement, moving away from the micropore. Approximate playback speed of 720x, with original video recorded over a 4-hour period. *Scale bar (10µm).*

**Figure S5. [Figure-S5 (Ye).mp4] Video shows live imaging of an SH-SY5Y cell on an 8µm pore diameter microporous membrane**. The SH-SY5Y cell moves away from a pore at the top of the frame, a neurite encounters a pore the right of the frame and retracts, and then the advancing edge of the cell encounters a pore at the bottom of the frame, at which point the cell changes direction, moving up the frame away from the edge of the micropore. Approximate playback speed of 720x, with original video recorded over a 4-hour period. *Scale bar (10µm).*

**Figure S6. [Figure-S6 (Ye).mp4] Video shows live imaging of an SH-SY5Y cell on an 8µm pore diameter microporous membrane**. The SH-SY5Y cell moves over a pore and encounters a set of pores at the top of the frame, at which point the cell changes direction, moving away from the edge of the micropores. Approximate playback speed of 720x, with original video recorded over a 4-hour period. *Scale bar (10µm).*

**Supplementary Methods**

**NT2/D1 neuron culture**

Human embryonal teratocarcinoma Ntera2/D1 (NT2/D1) cells were obtained from Professor Peter Andrews (Sheffield University, UK) and routinely cultured in Dulbecco’s modified Eagle medium with high glucose (4,500 mg/L) and stable glutamine (DMEM; PAA, UK), supplemented with 10% (v/v) heat inactivated FBS (Gibco, UK) and 1% PS. Media was exchanged every 2-3 days, and cells were split (1:3) when 80% confluent by mechanical scraping. NT2/D1 cells were differentiated in DMEM containing 5% FBS, 1% PS and 10µM RA, following a method similar to that described by Pleasure et al. [28]. Briefly, cells were exposed to RA for 4 weeks without passage, changing media every 2-3 days, after which cells were scraped, split into new flasks (1:3), and cultured for a further 2 days. Flasks were mechanically knocked ten times to dislodge cells, and the floating cells were reseeded into new tissue culture treated flasks and cultured for a further 2 weeks in DMEM media supplemented with 5% FBS, 1% PS and mitotic inhibitors (1µM cytosine arabinoside, 10µM fluorodexoyuridine, and 10µM uridine; Sigma-Aldrich, UK). At this point cells were mechanically detached from flasks and seeded directly onto treated and coated membranes. DMEM media with 2% (v/v) Neuronal Stem Cell Supplement (PAA, UK), and 1% PS was used to support the culture of the differentiated neurons on the porous membranes.

**Live cell imaging**

Images of SH-SY5Y neurons seeded at very low density (1x10^4^ cells/cm^2^) were cultured on treated membranes with 8µm diameter pores for 48 hrs and then inverted and placed into a glass bottomed petridish in culture medium. The dish was placed into a live imaging stage (OKOlab Stage Top Chamber, OKOlab, USA) and maintained at 37ºC with 5% CO2 in humidified air with the microscope setup as described above. Images were recorded using brightfield imaging and a 10x lens with phase contrast, at 1 frame/minute for up to 2 hrs. To highlight cells, false colour was added to selected images using CorelPhotoPaint X7 (Corel, UK).
